# Surveillance of medical resources for stroke in rural Japan through the Jichi medical university alumni network

**DOI:** 10.1016/j.neuros.2026.100062

**Published:** 2026-06-19

**Authors:** Takafumi Mashiko, Yuhei Anan, Kosuke Matsuzono, Tadashi Ozawa, Reiji Koide, Ryota Tanaka, Shigeru Fujimoto

**Affiliations:** Division of Neurology, Department of Medicine, Jichi Medical University, Yakushiji 3311-1, Shimotsuke, Tochigi 329-0498 Japan

**Keywords:** Telestroke, Medical resources, Rural area, Education

## Abstract

**Background:**

Japan’s rapidly aging population has made equitable delivery of stroke care a national priority. However, the status of medical resources in remote regions remains unclear.

**Methods:**

A questionnaire survey was distributed in 2017–2018 to the Jichi Medical University alumni network, whose members provide medical care in rural communities and outlying islands.

**Results:**

A total of 385 responses were received (response rate = 44.8%), and 236 responses from institutes in rural or island regions were analyzed. The median number of physicians per institute was two in rural areas and one on islands, with approximately 80% specializing in general internal medicine. Among 4569 patients with stroke or transient ischemic attack, including 2860 with ischemic stroke, 95 (3.3%) received intravenous tissue plasminogen activator and 27 (0.95%) underwent mechanical thrombectomy. Computed tomography was available in 116 institutes (49.2%), and carotid ultrasonography in 174 (73.7%). Antithrombotic therapy was available in approximately 60% of institutes. Of respondents, 185 of 201 (92.0%) in rural areas and 31 of 32 (96.9%) on islands recognized the need for stroke care education, while 132 (65.7%) and 25 (78.1%), respectively, expressed demand for telestroke support. However, telestroke systems were available in only 36 rural institutes (17.6%) and 13 islands (40.6%).

**Conclusions:**

This study reveals the substantial unmet need for stroke care resources in rural and remote settings. While the availability of physicians and imaging devices has partially improved, major challenges remain, including unequal access to reperfusion therapy, limited supply of essential medications, and insufficient telemedicine infrastructure.

## Introduction

Stroke is one of the leading causes of death and long-term disability in Japan [1], making the improvement and standardization of stroke care an urgent national priority in a country with a super-aged society [2]. For ischemic stroke, rapid administration of intravenous recombinant tissue plasminogen activator (IV-tPA) and/or timely mechanical thrombectomy (MT) is essential to reduce post-stroke dependence [3,4].

Continuing appropriate treatments, including antithrombotic therapy, is also crucial during the chronic phase to prevent recurrence [5]. In December 2018, Japan enacted the ‘Basic Plan to Promote Stroke and Cardiovascular Disease Control Programs (Stroke and Cardiovascular Disease Control Act),’which supports comprehensive stroke management nationwide [6]. Under this initiative, the Japan Stroke Society certifies two key types of facilities: primary stroke centers (PSCs), which accept patients with stroke 24 hours a day throughout the year, and PSC cores, which can perform MT continuously [7]. These centers provide intensive acute care for patients with stroke across designated medical regions [6].

Despite these national efforts, many rural and island regions continue to face shortages of physicians and medical devices [8,9]. In the United States, studies have reported that patients in rural areas had higher stroke incidence and mortality rates than those in urban areas, influenced by multiple factors such as poorer risk factor profiles, limited access to acute care, lower socioeconomic status, and reduced educational attainment [10–12]. Similarly, in Japan, IV-tPA administration rates have been associated with the local availability of stroke specialists, revealing disparities between rural and urban areas [13,14]. In such rural areas, patients with acute stroke may receive IV-tPA through telestroke support and subsequently be transferred to PSCs for MT, later returning to rural hospitals for ongoing care. However, the nature and extent of such bidirectional cooperation between rural facilities and PSCs remain unclear.

Jichi Medical University (JMU) was founded through a government-led program designed to address the shortage of physicians in Japan [15]. JMU recruits students from all 47 prefectures, (equivalent to a state in the US) offering full tuition waivers for its 6-year medical program in exchange for mandatory post-graduation service in rural or island communities for 6–7 years [16]. Although some alumni later relocate to urban regions, this program continues to maintain the provision of rural medical services nationwide.

This study aimed to assess available resources and unmet needs for stroke care in rural Japanese communities and outlying islands not directly covered by PSCs. Using a questionnaire survey distributed through the JMU alumni network—comprising physicians trained specifically for rural service—this study sought to identify practical strategies to optimize medical resource distribution and reduce regional disparities in stroke care delivery.

## Methods

Questionnaires were sent to all JMU alumni listed as working in rural areas and outlying islands. The survey included the following items (Supplementary Table 1): field of specialization; location (urban, rural, or island); type of institution (clinic, where up to nine patients can be admitted, or hospital); number of physicians; population served by each institute; and number of patients with stroke or TIA —categorized as intracerebral hemorrhage, subarachnoid hemorrhage, cerebral infarction (CI), TIA, and others—between April 2017 and March 2018. Additional items included the number of patients with CI arriving within 4.5 hours and receiving reperfusion therapy, transportation methods and travel times to comprehensive hospitals, availability of antithrombotic agents and diagnostic devices, capacity for PT-INR testing, and availability or demand for telestroke systems and stroke education activities. Responses from urban institutes and duplicates from overlapping institutes were excluded.

Geographic locations of the participating institutions were determined based on their names and postal codes. Each institution was individually searched using Google Maps to obtain latitude and longitude coordinates. Administrative boundary data for Japan (2018 edition) were obtained from the Geospatial Information Authority of Japan [17]. A nationwide map delineated by prefectures was generated using QGIS (Long Term Release, version 3.44.9), and the locations of the institutions were subsequently plotted.

Ethical approval was not required because this study involved an anonymous questionnaire survey of healthcare professionals and did not include any patient data or personally identifiable information, in accordance with institutional guidelines.

### Statistical methods

Data were cleaned, merged, and analyzed centrally using JMP Version 14.2 (SAS Institute Inc., Cary, NC, USA).

## Results

**Fig. 1.**
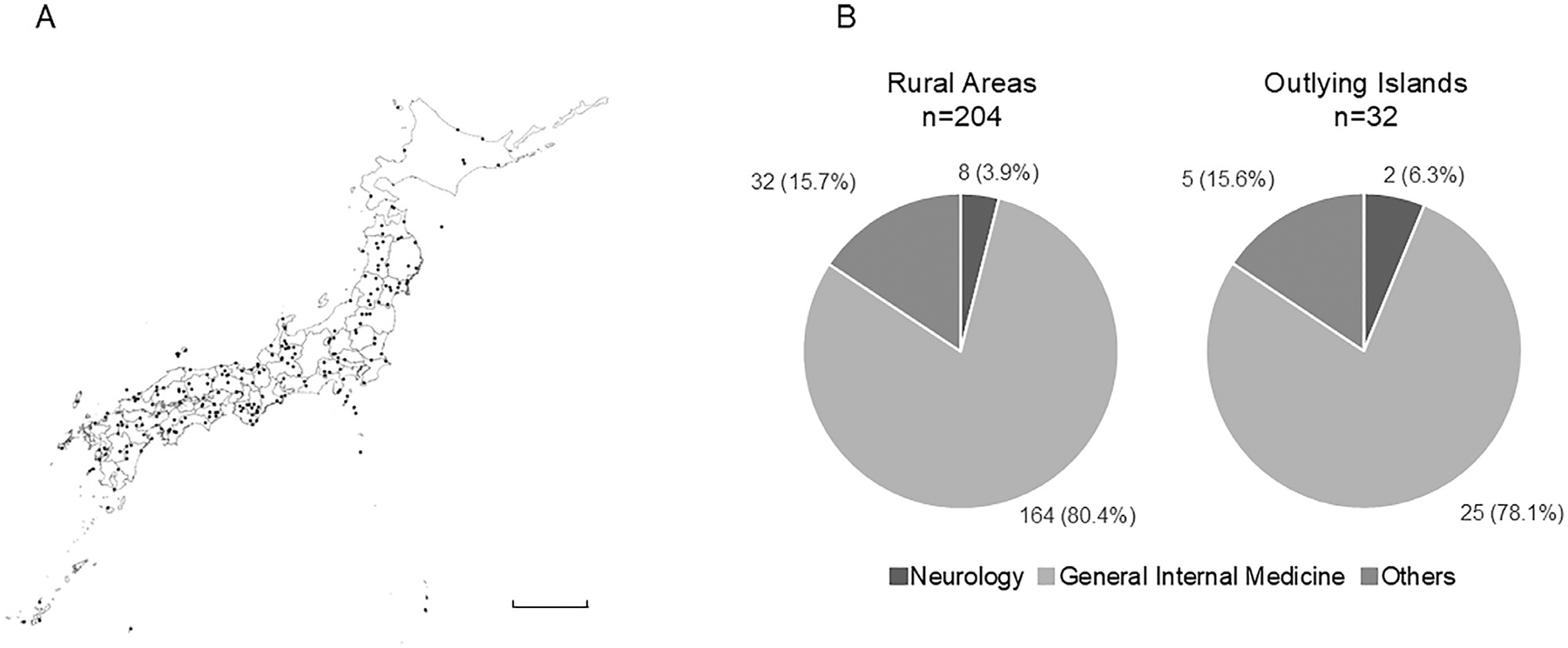
(A) Geographic distribution of responding facilities in rural areas and outlying islands across Japan. This map of Japan, shown at the prefectural level, plots the locations of facilities in rural areas and outlying islands from which survey responses were obtained. The scale bar in the lower right indicates 250 km. (B) Proportion of physicians by declared medical specialty in rural areas and on outlying islands.

A total of 859 questionnaires were distributed, and 385 were returned (response rate = 44.8%). After excluding 149 responses from urban or overlapping institutes, 236 responses were analyzed. As shown in Fig. 1A, these 236 facilities were broadly distributed throughout Japan, including remote islands. Of these, 204 (86.4%) were from rural areas and 32 (13.6%) from outlying islands. The median number of physicians per institute was two (interquartile range [IQR]: 1–6) in rural areas and one (IQR: 1–3) at on islands. Fig. 1B shows the distribution of physician specialties in each institute. General internal medicine was the predominant specialty—164 (80.4%) in rural areas and 25 (78.1%) on islands—while neurologists and neurosurgeons accounted for only eight (3.9%) and two (6.3%), respectively.

### Characteristics of patients with stroke in rural areas and islands

**Fig. 2.**
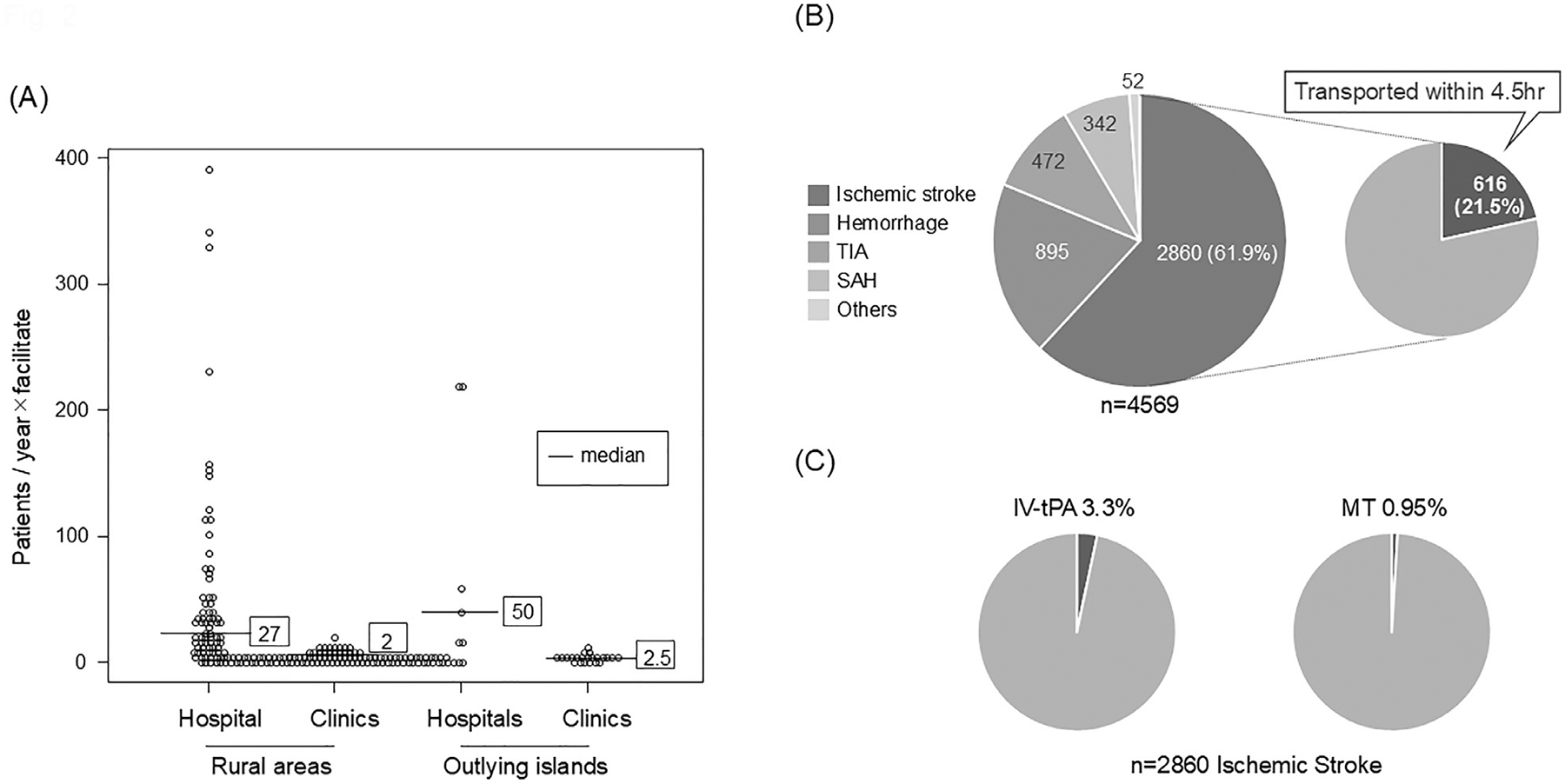
(A) Distribution of the annual number of patients with stroke treated per facility (hospitals and clinics in rural areas and on outlying islands). The horizontal line indicates the median number of patients. (B) Proportion of stroke subtypes among the total number of patients with stroke across all facilities within 1 year, and the proportion of patients with ischemic stroke transported within 4.5 hours of onset. (C) Proportion of patients with ischemic stroke who received intravenous thrombolysis (IV-tPA) and those who underwent endovascular thrombectomy (EVT).

Fig. 2A illustrates the distribution of annual stroke and TIA incidence in each institute, and stroke subtypes across rural areas and islands. In total, 4569 patients with stroke or TIA were reported, including 2860 (61.9%) patients with ischemic stroke. Among these, 616 (21.5%) arrived at local institutes within 4.5 hours (Fig. 2B). Of 4569 patients with stroke or TIA, 680 were transferred to core hospitals, 95 (3.3% received IV-tPA, and 27 (0.95%) underwent MT (Fig. 2C).

**Fig. 3.**
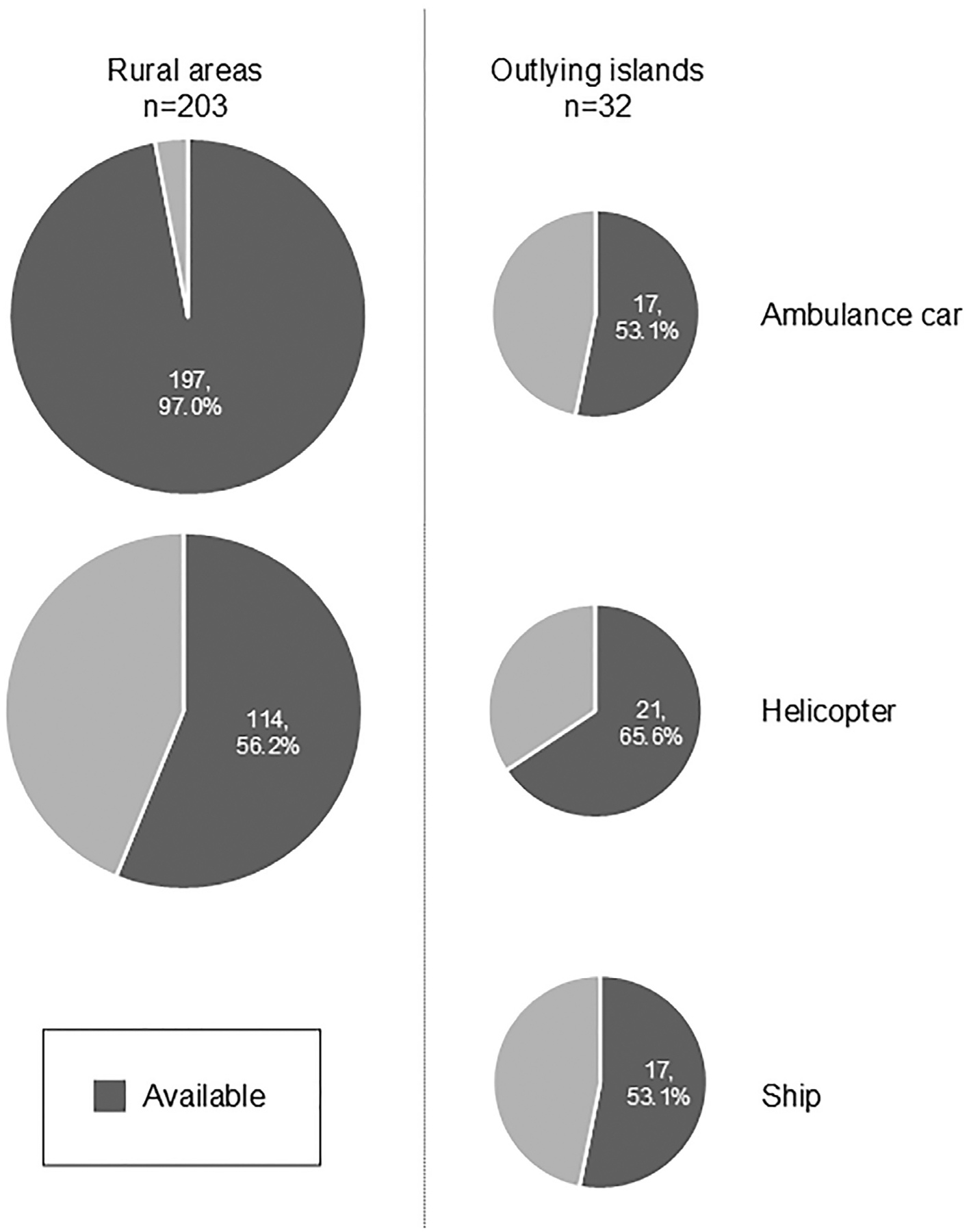
Responses regarding available patient transportation methods (ambulance, helicopter, and ship) in rural areas and on outlying islands.

Among 203 institutes in rural areas, 197 (97.0%) used public ambulances and 114 (56.2%) used helicopters. On outlying islands, 17 of 32 institutes (53.1%) used ambulances, 21 (65.6%) used helicopters, and 17 (53.1%) used ships (Fig. 3). The median time to departure was 30 min (IQR, 15–30) in rural areas and 30 min (IQR, 15–60) in outlying islands. The median time to reach their destination was 30 min (IQR, 20–45) in rural areas and 60 min (IQR, 20–70) in outlying islands.

### Medical supply conditions for stroke in rural areas and islands

Computed tomography (CT) scans were available at 116 institutes (49.2%), and MRI was available at 61 institutes (25.8%). Carotid sonography was accessible at 174 institutes (73.7%). The distribution of institutions based on same-day testing capability for PT-INR measurement, which is essential for warfarin therapy, was also assessed. Among hospitals, immediate (same-day) PT-INR testing was available at nearly all institutes, including 80 institutes (97.6%) in rural areas and eight (100%) on outlying islands. In contrast, among clinics, immediate testing was available at 20 institutions (83.3%) on outlying islands, with 3 institutes (12.5%) able to obtain results by the next day. In rural areas, 56 institutes (46.2%) could perform immediate testing; 35 institutes (28.9%) reported results the next day, 25 institutes (20.7%) required two or more days, and 5 institutes (4.1%) could not perform PT-INR testing.

**Fig. 4.**
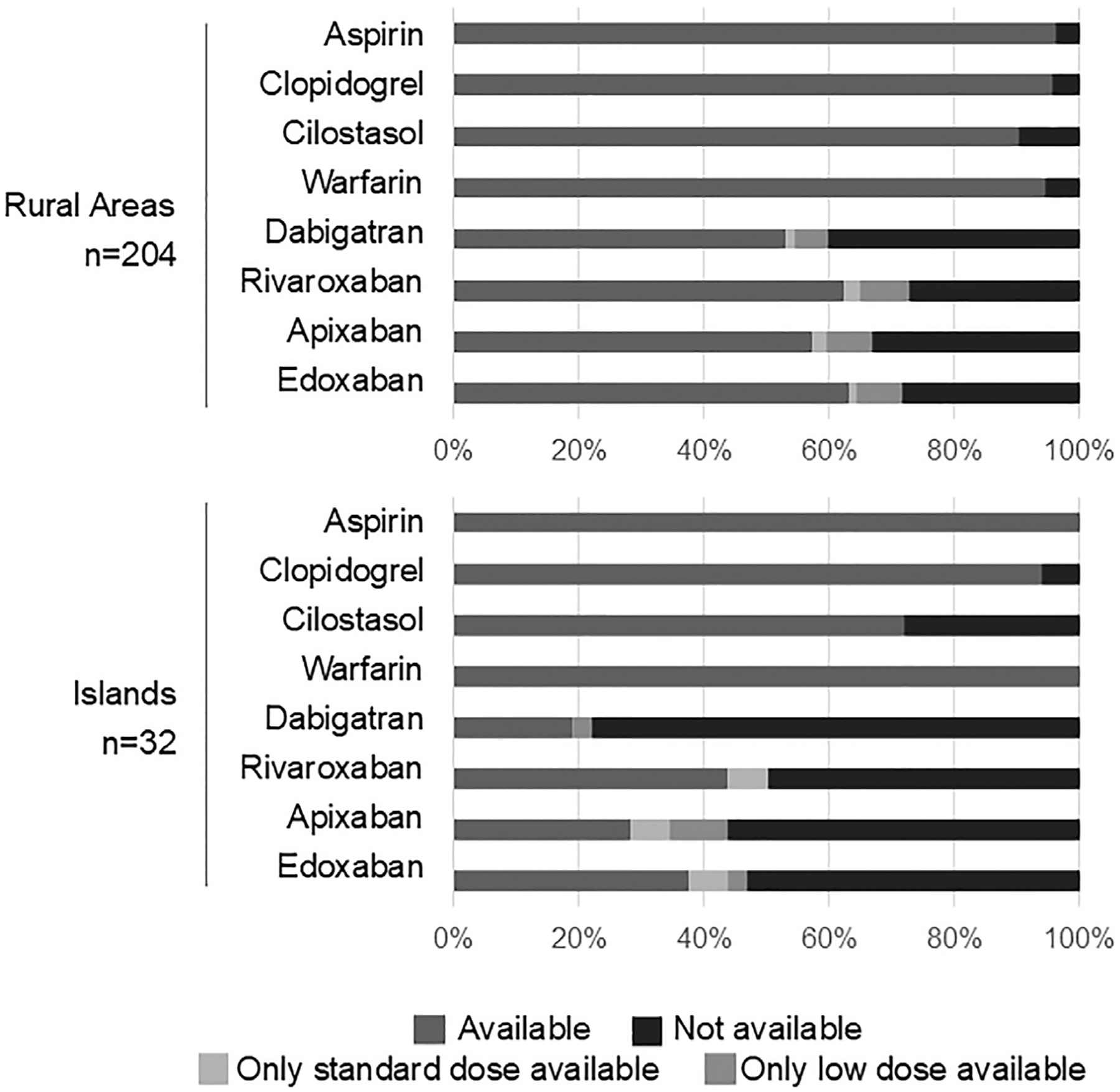
Proportion of facilities in rural and island regions with each antithrombotic agent available.

Fig. 4 illustrates the availability of anti-thrombotic agents. Commonly used anti-platelet agents such as aspirin and clopidogrel were available at nearly all institutes (aspirin at 196 institutes [96.1%] in rural areas and 32 [100%] on islands; clopidogrel at 195 [95.6%] and 30 [93.8%], respectively). However, newer anti-platelet agents such as cilostazol (184 [90.2%] in rural areas and 23 [71.9%] on islands) and prasugrel (65 [31.9%] and 9 [28.1%]) were available at fewer institutions.

Regarding anti-coagulant agents, warfarin was available at nearly all institutes (94.6% in rural areas and 100% on islands). However, there were differences in the availability of both standard and low doses of DOACs. For dabigatran, both doses were available at 108 institutes (52.9%) in rural areas and six (18.8%) on islands; only the standard dose was available at three (1.5%) and 0 (0%), and only the low dose at 11 (5.4%) and one (3.1%), respectively. For rivaroxaban, both doses were available at 127 institutes (62.3%) and 14 (43.8%), only the standard dose at five (2.5%) and two (6.3%), and only the low dose at 16 (7.9%) and 0, respectively. For apixaban, both doses were available at 117 institutes (57.4%) and nine (28.1%), only the standard dose at four (2.0%) and two (6.3%), and only the low dose at 15 (7.4%) and three (9.4%). For edoxaban, both doses were available at 129 institutes (63.2%) and 12 (37.5%), only the standard dose at two (1.0%) and two (6.3%), and only the low dose at 15 (7.4%) and one (3.1%), respectively.

### Incentive for stroke care in rural areas and islands

**Fig. 5.**
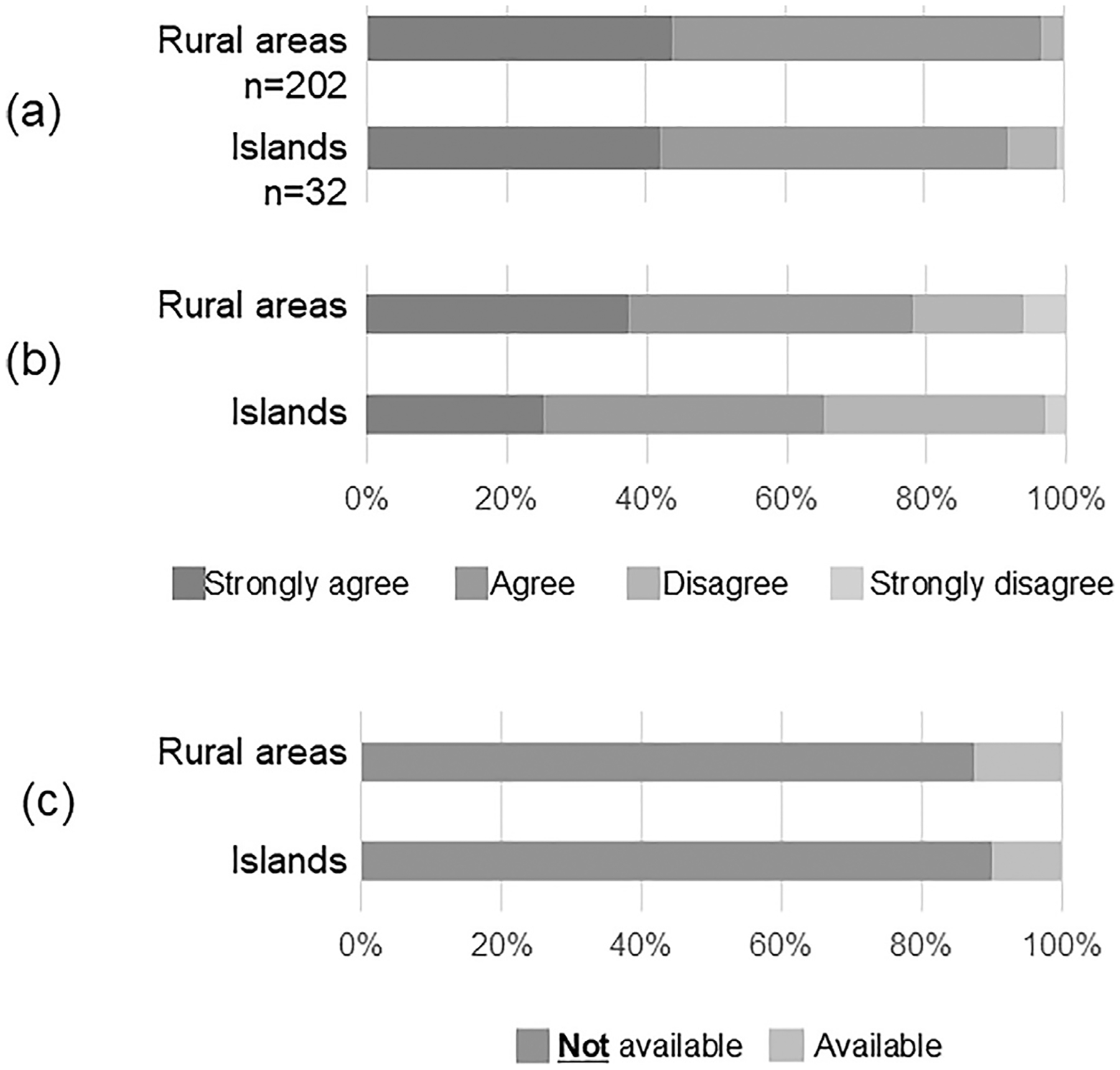
Proportion of respondents who perceived the need for stroke awareness activities in rural areas and on outlying islands. (A) Percentage of respondents who perceived the need for awareness activities for stroke care. (B) Percentage of respondents who perceived the need for the remote telestroke support systems. (C) Percentages of respondents whose institutes or regions currently have awareness programs.

Telestroke services were available at 36 institutes (17.6%) in rural areas and 13 (40.6%) on islands. Fig. 5 shows the demand for stroke awareness activities in these regions. A majority—185 of 201 (92.0%) in rural areas and 31 of 32 (96.9%) on islands—recognized the need for public education on stroke care. Additionally, 132 (65.7%) in rural areas and 25 (78.1%) on islands expressed the need for remote telestroke support systems. However, 183 of 203 (90.2%) in rural areas and 28 of 32 (87.5%) on islands reported no existing awareness programs in their institutes or regions.

## Discussion

Through a questionnaire survey on medical resources for stroke in Japanese rural areas and outlying islands, conducted via the JMU alumni network, it became evident that there is substantial demand for stroke treatment in these regions. The findings highlighted concerns regarding reperfusion therapy, limitations in the medical supply system, disparities in drug availability, underdeveloped remote medical infrastructure, a heavy clinical burden on non-stroke specialists providing acute care, and inadequate medical education systems to address these needs.

Among the 55 facilities listed in the survey, several reported an incidence rate of 0, suggesting that they might not manage stroke cases directly but instead focus on other medical conditions within their service areas. The existence of numerous facilities reporting no stroke cases, even in densely populated regions, supports this assumption. Although 55 facilities reported zero stroke patients yearly, nearly half of the hospitals—regardless of whether they were located in rural or island areas—treated more than 25 patients with stroke annually. Furthermore, most clinics managed at least two stroke cases annually, indicating a strong demand for stroke care services. This pattern suggests regional disparities in healthcare resource allocation, as well as a concentration of acute stroke cases in specific institutions. In such institutions, a limited number of non-specialists appear to manage a large number of stroke patients. Many of these non-specialists expressed a need for stroke education initiatives and telestroke support. The aging population in remote regions may further contribute to this situation.

Regarding acute ischemic stroke, the survey revealed that even in rural and island regions, a considerable number of patients were transported within 4.5 hours of onset, making them eligible for reperfusion therapy. However, the implementation rate of reperfusion therapy was significantly lower than the approximately 16% reported in Japan’s stroke database [18]. Although PSCs have been established and various transportation systems have developed, the consistent delivery of reperfusion therapy to eligible patients remains challenging. While this survey did not identify specific reasons for the low implementation rate, remote stroke care through telestroke may serve as a viable solution. Telestroke—using information and communication technology (ICT) to allow stroke specialists to provide remote support—can enable safe and effective reperfusion therapy [19–22], optimizing the use of medical resources [23,24].

However, the role of telestroke in Japan may differ somewhat from that in Western countries, particularly the United States, where telestroke has already been implemented on a much larger scale; thus, direct quantitative comparisons may not be straightforward. In the United States, where approximately 20% of the population lives in rural areas across a vast geographic expanse [25], telestroke—and telemedicine more broadly—has been regarded primarily as a means of overcoming absolute distance and improving equity in access to stroke care. Mobile stroke units represent one example of this approach [26]. In contrast, Japan has a smaller land area, much of it mountainous, and most of its population is concentrated in limited plains and urban areas. Accordingly, PSCs have been reported to cover 98.9% of the population (unpublished data). In this context, the expected role of telestroke in Japan is not simply to bridge long distances, but rather to deliver stroke expertise to residents in mountainous regions, remote communities, and outlying islands that remain beyond the practical reach of existing stroke care systems. In this sense, the present findings reflect the real-world status of stroke care in precisely such underserved areas.

Encouragingly, the survey demonstrated that CT, an essential imaging modality for acute stroke assessment and IV-tPA eligibility evaluation, is widely available in both rural and island regions. Carotid sonography was also available in many facilities; although it is not required for IV-tPA administration and does not replace CT angiography for comprehensive vascular evaluation, it may serve as a supplementary, low-invasive tool for initial vascular screening or triage support in resource-limited remote settings. As part of national health initiatives, telestroke-based support for reperfusion therapy has now been incorporated into reimbursement schemes. Promoting telestroke is therefore crucial [27], accompanied by community awareness campaigns to educate residents about IV-tPA and MT [28]. The Japan Stroke Society has established telestroke guidelines continues to strengthen its promotional efforts [29]. Since 2024, telestroke-based administration of IV-tPA and MT eligibility assessments in government-designated rural and island areas have been covered under national insurance in accordance with these guidelines. Consequently, stroke specialists and PSCs are expected to play a central role in ensuring the successful implementation of reperfusion therapy. Ongoing advancement in medical education systems is anticipated to further enhance these efforts.

Even within the acute phase of ischemic stroke, there is an approach known as “Drip & Stay,” in which conservative therapy is provided following IV-tPA administration without MT, particularly for patients with mild cases [30,31]. In such situations, rigorous risk management and the continued, evidence-based use of antithrombotic agents are essential.

One major concern identified in this study is the uneven availability of DOACs. Although DOACs are manufactured in two or three dosage options, it is not uncommon for rural or island regions to have access to only one dosage form. Because DOAC dosages are determined according to strict evidence-based criteria, inappropriate dosage use has been associated with poorer patient outcomes [32]. Therefore, addressing regional disparities in drug distribution is crucial to ensuring proper clinical use.

In regions where DOACs are unavailable, anticoagulation therapy for conditions such as non-valvular atrial fibrillation typically depends on warfarin. However, achieving therapeutic efficacy comparable to that of DOACs requires consistent PT-INR monitoring [33,34]. The inability to perform real-time PT-INR testing remains a critical unresolved issue, particularly among rural clinics, and poses a challenge to providing safe and effective anticoagulation therapy.

### Limitations

This study has several limitations. First, the questionnaire survey targeted a limited cohort—members of the JMU alumni network. Although this university was founded to train healthcare professionals who would support regional healthcare, the network may not fully represent all aspects of regional healthcare delivery across Japan. Second, because the data were obtained through self-administered questionnaires, the reliability of the responses depends on the respondents, whose shared background as JMU graduates is the only basis for credibility. Third, the data were collected between 2017 and 2018, meaning that significant time has passed since the survey. The findings do not reflect the potential impact of three major events that have since occurred in Japan: the COVID-19 pandemic (2021–2023), the implementation of physician work-style reforms in April 2024, and the inclusion of remote medical treatment reimbursement for stroke-related care in April 2024. Each of these developments may have influenced the stroke care environment. Therefore, a follow-up survey conducted in the current context is warranted. Fourth, although the questionnaire included information on average daily outpatient volume, we did not perform subgroup analyses according to facility volume. The primary purpose of this study was to describe the overall status of stroke care in rural areas and outlying islands in Japan rather than to assess differences between higher- and lower-volume facilities. However, facility volume may influence transfer patterns, diagnostic capacity, and the use of reperfusion therapies. Therefore, future studies incorporating such subgroup analyses may provide additional insights.

## Conclusions

This survey, conducted through the JMU alumni network, revealed a substantial demand for acute stroke treatment in Japan’s rural and island regions. While some human and material resources are available, notable challenges remain—particularly regional disparities in reperfusion therapy implementation and limited availability of essential medications. Therefore, the continued development of telestroke systems and the enhancement of medical education programs are critical. The findings of this research may serve as valuable references for policy formulation and strategic planning aimed at strengthening regional healthcare systems and improving equitable access to stroke treatment.

## Supplementary Material

Supplementary Material

Supplementary material associated with this article can be found, in the online version, at https://doi.org/10.1016/j.neuros.2026.100062.
