# Supplementary material for: Surveillance of medical resources for stroke in rural Japan through the Jichi medical university alumni network
Source: Equity Neurosci. Author manuscript; Available in PMC 2026 Jul 24. (PMC13390898; doi:10.1016/j.neuros.2026.100062)
Supplement: Supplementary Material [file NIHMS2194188-supplement-Supplementary_Material.docx]

**Supplementary Data**

Surveillance of Medical Resources for Stroke in Rural Japan through the Jichi Medical University Alumni Network

Takafumi Mashiko, MD, PhD, MBA, Yuhei Anan, MD, PhD, Kosuke Matsuzono, MD, PhD, Tadashi Ozawa, MD^,^ PhD, Reiji Koide, MD, PhD, Ryota Tanaka, MD, PhD, Shigeru Fujimoto, MD, PhD

**Table of contents**

**S-2~5. Supplementary Table 1**

**S-6. Abbreviations**

Supplementary Table 1. Questionnaire on Stroke Care

Original Japanese and English Translation

| Q No. | 日本語原文（Original Japanese） | 英語訳（English Translation） |
| --- | --- | --- |
| 0 | 先生の専門または所属の診療科は何科ですか． | Specialty / Department |
| 1 | 勤務されているご施設はどれですか．  a. 僻地の診療所  b. 離島の診療所  c. 僻地の病院  d. 離島の病院  e. その他（　　　　　　　　　） | Type of facility  a. Rural clinic  b. outlying island clinic  c. Rural hospital  d. Outlying island hospital  e. Other (specify) |
| 2 | ご勤務の施設はあなたを含めて何人の医師で診療していますか． | Number of physicians at your facility |
| 3 | ご勤務の施設では1日平均何人の外来患者さんが来院されますか．  a. 10人未満  b. 10〜19人  c. 20〜29人  d. 30〜49人  e. 50人以上 | Average daily outpatients  a. <10  b. 10–19  c. 20–29  d. 30–49  e. ≥50 |
| 4 | 2017年4月から2018年3月までに発症7日以内の脳卒中急性期患者または一過性脳虚血発作患者は何人いましたか．  合計（　）人  脳出血（　）人  くも膜下出血（　）人  脳梗塞（　）人  一過性脳虚血発作（　）人  詳細不明（　）人 | Number of patients presenting within 7 days of stroke onset or with TIA (Apr 2017 – Mar 2018)  Total: n= ___  Intracerebral hemorrhage: n= ___  Subarachnoid hemorrhage: n= ___  Ischemic stroke: n= ___  Transient ischemic attack (TIA): n= ___  Unspecified: n= ___ |
| 5 | その脳梗塞の中で発症4.5時間以内の患者は何人いましたか． | Among ischemic stroke patients, number presenting within 4.5 h of onset |
| 6 | 急性期脳卒中患者が来院した場合，搬送先の病院は決まっていますか．  a. 脳卒中専門医がいる病院  b. 脳卒中専門医がいない救急病院  c. 脳卒中専門医の存在が不明な救急病院  d. 特定の病院は決まっていない | Predefined destination hospital for acute stroke  a. Hospital with stroke specialist  b. Emergency hospital without stroke specialist  c. Emergency hospital (specialist availability unknown)  d. No predefined hospital |
| 7 | Q4の患者のうち何人を搬送しましたか． | Number of patients transferred (from Q4) |
| 8 | 遠隔医療サポートのシステムはありますか．  a. ある（内容：　）  b. ない | Telemedicine support system available  a. Yes (specify)  b. No |
| 9 | 患者を搬送する場合の搬送方法はどれですか．(複数選択可)  a. 救急車  b. ドクターヘリ  c. 船  d. 施設所有の搬送車  e. その他 | Transfer methods (select all that apply)  a. Ambulance  b. Doctor helicopter  c. Boat  d. Facility-owned transport  e. Other (specify) |
| 10 | Q9でb〜eと回答した場合、その搬送手段はいつでも利用可能ですか．  a. 24時間利用可能  b. 平日日中のみ  c. 平日のみ  d. 平日休日の日中のみ  e. その他 | Availability of methods selected in Q9 (b–e)  a. 24 h  b. Weekday daytime only  c. Weekdays only  d. Daytime only (weekdays & weekends)  e. Other (specify) |
| 11 | 搬送にかかる最短時間を教えてください．  自施設来院から出発まで（　）分  自施設出発から搬送先到着まで（　）分 | Minimum transfer time  Arrival → departure: ___ min  Departure → destination: ___ min |
| 12 | Q5の患者で、搬送先でrt-PA静注療法ないし血栓回収療法を受けた症例は何例ですか．  rt-PA静注療法（　）人  血栓回収療法（　）人 | Reperfusion therapy at destination (among Q5)  IV-tPA: n= ___  Mechanical thrombectomy: n= ___ |
| 13 | 搬送した患者のうち何人が自宅に戻れましたか． | Number of transferred patients who returned home |
| 14 | ご勤務の施設では脳卒中についての市民啓発プログラムはありますか．  a. 施設でのプログラムがある  b. 施設にはないが地域のプログラムがある  c. 特にプログラムはない | Public awareness program on stroke  a. Provided at facility  b. Community program only  c. None |
| 15 | 脳卒中予防や発症時の対処法などの市民啓発の必要性を感じますか．  a. 強く感じる  b. どちらかといえば感じる  c. あまり感じない  d. 全く感じない | Perceived necessity of public education on stroke prevention and acute management  a. Strongly agree  b. Agree  c. Disagree  d. Strongly disagree |
| 16 | 市民啓発の資材や人材を提供された場合，利用されたいと思いますか．  a. 是非利用したい  b. 利用してみてもいい  c. 必要ない | Willingness to use provided educational resources  a. Definitely yes  b. Possibly yes  c. Not necessary |
| 17 | 脳卒中急性期の遠隔診療サポートは必要と思われますか．  a. 強く感じる  b. どちらかといえば感じる  c. あまり感じない  d. まったく感じない | Perceived necessity of telemedicine support  a. Strongly agree  b. Agree  c. Disagree  d. Strongly disagree |
| 18 | ご勤務の施設で使用可能な抗血栓薬を選んでください．  a. アスピリン  b. クロピドグレル  c. シロスタゾール  d. プラスグレル  e. ワルファリン  f. ダビガトラン（高/低/両方）  g. リバーロキサバン（高/低/両方）  h. アピキサバン（高/低/両方）  i. エドキサバン（高/低/両方） | Antithrombotic therapies available  a. Aspirin  b. Clopidogrel  c. Cilostazol  d. Prasugrel  e. Warfarin  f. Dabigatran (high/low/both)  g. Rivaroxaban (high/low/both)  h. Apixaban (high/low/both)  i. Edoxaban (high/low/both) |
| 19 | ご勤務の施設で施行可能な検査をすべて選んでください．  a. 頭部CT  b. 頭部MR  c. 造影CT angiography  d. 頸部血管エコー  e. 経胸壁心臓エコー  f. 経食道心臓エコー  g. 経頭蓋ドプラまたはカラー  h. Holter心電図  i. 植込み型心電計 | Diagnostic tests available  a. Head CT  b. Brain MRI  c. Contrast CT angiography  d. Carotid ultrasound  e. Transthoracic echo  f. Transesophageal echo  g. Transcranial Doppler/color Doppler  h. Holter ECG  i. Implantable loop recorder |
| 20 | ご勤務の施設でのPT-INRの検査状況を教えてください．  a. 即日検査結果を確認可能  b. 翌日検査結果を確認可能  c. 翌日以降に検査結果を確認可能  d. 検査不可 | Turnaround time for PT-INR testing  a. Same day  b. Next day  c. ≥2 days  d. Not available |
| 21 | 一過性脳虚血発作患者が受診された時の対応について教えてください．  a. ご施設で即日検査＋投薬  b. 検査予約＋暫定投薬  c. 検査予約＋結果に基づき投薬選択  d. 即日専門病院紹介  e. 後日専門病院紹介  f. 暫定投薬＋即日紹介  g. 暫定投薬＋後日紹介  h. その他 | Management of TIA patients  a. Same-day test + start therapy  b. Schedule test + provisional therapy  c. Schedule test + select therapy based on result  d. Immediate referral  e. Later referral  f. Start provisional therapy + immediate referral  g. Start provisional therapy + later referral  h. Other (specify) |
| 22 | ご勤務の施設，ご記入いただいた先生のお名前，卒業年  郵便番号（　　　）  施設名  診療科  お名前  卒業年 | Facility and physician details  Postal code: ___  Facility name  Department  Name  Year of graduation |

Abbreviations

CT: Computed Tomography

ECG: Electrocardiogram

IV-tPA: Intravenous recombinant tissue plasminogen activator

MRI: Magnetic Resonance Imaging

PT-INR: Prothrombin Time - International Normalized Ratio

TIA: Transient Ischemic Attack
